# Evaluating Joint Morbidity after Chondral Harvest for Autologous Chondrocyte Implantation (ACI): A Study of ACI-Treated Ankles and Hips with a Knee Chondral Harvest

**DOI:** 10.1177/1947603515607963

**Published:** 2016-01

**Authors:** Helen S. McCarthy, James B. Richardson, Jane C. E. Parker, Sally Roberts

**Affiliations:** 1 Centre for Spinal Studies, Robert Jones and Agnes Orthopaedic Hospital NHS Foundation Trust, Oswestry, Shropshire, UK; 2 ISTM, Keele University, Keele, Staffordshire, UK; 3 Institute of Orthopaedics, Robert Jones and Agnes Hunt Orthopaedic Hospital NHS Foundation Trust, Oswestry, Shropshire, UK

**Keywords:** autologous chondrocyte implantation, cartilage repair, donor-site morbidity, osteoarthritis, histology

## Abstract

**Objective:**

To establish if harvesting cartilage to source chondrocytes for autologous chondrocyte implantation (ACI) results in donor site morbidity.

**Design:**

Twenty-three patients underwent ACI for chondral defects of either the ankle or the hip. This involved cartilage harvest from the knee (stage I), chondrocyte expansion in the laboratory and implantation surgery (stage II) into the affected joint. Prior to chondral harvest, no patient had sought treatment for their knee. Lysholm knee scores were completed prior to chondral harvest and annually post-ACI. Histological analyses of the donor site were performed at 12.3 ± 1.5 months for 3 additional patients who had previously had ACI of the knee.

**Results:**

The median preoperative Lysholm score was 100, with no significant differences observed at either 13.7±1.7 months or 4.8±1.8 years postharvest (median Lysholm scores 91.7 and 87.5, respectively). Patients whose cartilage was harvested from the central or medial trochlea had a significantly higher median Lysholm score at latest follow-up (97.9 and 93.4, respectively), compared with those taken from the intercondylar notch (median Lysholm score 66.7). The mean International Cartilage Repair Society (ICRS) II histological score for the biopsies taken from the donor site of 3 additional knee ACI patients was 117 ± 10 (maximum score 140).

**Conclusions:**

This study suggests that the chondral harvest site in ACI is not associated with significant joint morbidity, at least up to 5 years postharvest. However, one should carefully consider the location for chondral harvest as this has been shown to affect knee function in the longer term.

## Introduction

Chondral and osteochondral lesions in the hyaline cartilage of an articulating joint often require surgical intervention due to the limited ability of cartilage self-repair. Many techniques to restore articular cartilage and joint function have been established over the years, including mosaicplasty, osteochondral autograft transport system (OATS) and autologous chondrocyte implantation (ACI). Mosaicplasty and OATS involve obtaining cylindrical osteochondral grafts of varying sizes from the lesser loaded areas of the articular surface of the donor joint and implanting them into the recipient osteochondral lesion. Traditionally, mosaicplasty requires multiple small osteochondral grafts (typically 3-8 mm),^1-3^ whereas OATS usually requires one single large defect-sized graft (typically ~10 mm)^4^ and either procedure can be performed in a single operation. ACI on the other hand is a 2-stage procedure, requiring a chondral harvest of macroscopically healthy cartilage, also from a lesser loaded area of the joint (stage I) and subsequent chondrocyte isolation, culture, and proliferation. The resulting cells are then implanted into the defect (stage II) approximately 3 to 4 weeks later,^5^ beneath either a periosteal or collagen membrane such as ChondroGide.

Originally developed to treat chondral lesions in the knee,^5^ ACI has since been adapted for the treatment of chondral defects in other joints such as the ankle^6-9^ and less commonly, the hip.^10-13^ While encouraging results in terms of cartilage growth and improvement of joint function in the treated joint have been reported, little has been published on the potential morbidity caused by harvesting healthy tissue from the joint in ACI. What has been described regarding donor site morbidity in the literature to date predominantly relates to osteochondral harvest for mosaicplasty^3^ or OATS,^4,14^ which has obvious differences in potential issues for donor site morbidity than solely a chondral harvest due to the depth and size of the harvest taken. Donor site morbidity is an understandable concern as the integrity of the healthy, intact hyaline cartilage is violated.

In this study, we aimed to assess the effect of chondral harvest on knee function by looking at a small population of patients who had ACI treatment of either the ankle or the hip but with a chondral harvest obtained from the knee. This group of patients therefore provide a unique opportunity to assess the effect of controlled chondral injury on knee function. We have also analyzed the repair tissue formed at the donor site from 3 additional patients who previously underwent ACI for the knee.

## Method and Materials

### Ankle and Hip ACI patients

#### Patients and Surgical Technique

Between 1998 and 2009, 23 patients (16 males, 7 females; mean age 38.4 ± 10.1 years, range 17.2-61.3 years) underwent ACI treatment for chondral defects of the ankle (n = 19) and hip (n = 4), respectively. The average defect size treated was 2.1 ± 1.6 cm^2^ (range, 0.5-7.8 cm^2^). ACI surgery involved chondrocyte harvest at the knee (stage I) from the ispilateral side in 21 patients and the contralateral side for 1 ankle and 1 hip patient, followed by chondrocyte implantation surgery (stage II). Chondral harvest was performed arthroscopically through standard anterolateral and anteromedial portals under tourniquet control. Specimens of cartilage (mean weight 271.9 ± 97.6 mg, range 103-520 mg) were taken using a 5-mm gouge from a region of the knee with low weightbearing status. The biopsy site locations were recorded and the biopsy material was transported to our on-site Good Manufacturing Practice (GMP) standard “Oscell” cell manufacturing facility for chondrocyte culture. Ten samples were taken from the central trochlea (mean age 36.0 ± 14.0 years, range 17.2-61.3 years), 2 from the lateral trochlea (mean age 44.6 ± 1.6 years, range 43.5-45.8 years), 6 from the medial trochlea (mean age 40.7 ± 5.6 years, range 31.9-47.2 years), and 5 from the intercondylar notch (mean age 38.0 ± 6.1 years, range 28.3-44.3 years). A mean yield of 5.2 × 10^5^ ± 1.3 × 10^5^ (range 3 × 10^5^ to 8 × 10^5^) chondrocytes were obtained from the harvest biopsy. Following cell culture, a mean of 4.9 × 10^6^ ± 1.9 × 10^6^ chondrocytes (range 1.3 × 10^6^ to 8 × 10^6^) were implanted into the treatment site approximately 3 weeks later under either a periosteal (n = 20) or ChondroGide (n = 3) patch. One ankle patient required a follow-up knee arthroscopy 1.4 years postharvest. Patients in this study have been investigated as part of an ethically approved project (REACT 09/H1203/90, granted by West Midlands National Research Ethics Service).

Prior to chondral harvest, no patient had sought treatment for their knee. Modified Lysholm scores, a measure of knee function,^15^ were completed preoperatively and at yearly intervals post–cell implantation. Lysholm scores were categorized as excellent (95-100), good (84-94), fair (65-83), or poor (≤64).^16^

#### Statistical Analysis

A *post hoc* power analysis demonstrated a power of 0.8, at a significance level of 5% (*P* = 0.05) to identify a Lysholm score difference of 13 points,^19^ indicating that this study would require a paired sample of at least 10 patients. Data were tested for normality using the Shapiro-Wilk normality test. Nonparametric paired data (Lysholm scores) were analyzed for statistical differences with the Wilcoxon signed-rank test. Nonparametric unpaired data were analyzed for statistical differences using the Mann-Whitney *U* test. Correlations were tested for using a Spearman’s rank correlation. Statistical differences between grouped frequency data of the Lysholm score parameters were tested for using a chi-square test of independence. A *P* value of <0.05 was deemed significant. All statistical analyses were performed using the software program Analyse-it Software Ltd, Leeds, UK.

### Additional Donor Site Study from 3 Knee ACI Patients

#### Histology

Three additional patients, who had received ACI treatment for chondral defects in their knees, underwent a follow-up arthroscopy at 12.3 ± 1.5 months, with full informed consent. The donor site was examined and core biopsies of the repair tissue formed were obtained. These were snap-frozen in liquid nitrogen–cooled hexane prior to sectioning and 7-µm thick cryosections were collected onto poly-l-lysine–coated slides. Sections were stained with either hematoxylin and eosin (H&E) or toluidine blue (TB) and viewed with bright light microscopy to assess the general morphology and proteoglycan content of the repair tissue, respectively. Sections were also viewed under polarised light to determine collagen fibril orientation to distinguish between hyaline cartilage and fibrocartilage morphologies. Sections were scored using both the International Cartilage Repair Society (ICRS) II^17^ (maximum score 140) and the OsScore^18^ (maximum score 10) scoring systems, where a higher score for either systems represents a better quality of repair tissue.

#### Immunohistochemistry

In addition, immunohistochemistry for collagen types I and II was undertaken; for this cryosections were incubated with hyaluronidase prior to fixing in 4% formaldehyde. Antibodies against collagen type I (1:500, clone I-8H5, MP Biomedicals, Cambridge, UK) or type II (1:10, clone CIIC1, Developmental Studies Hybridoma Bank, IA, USA) were incubated for 60 minutes prior to the secondary goat anti-mouse biotinylated antibody for 60 minuntes (Vectastain Elite ABC kit, Vector Laboratories, Peterborough, UK). Adjacent sections were stained with an isotype-matched murine IgG1 (Dako, Cambridge, UK) as a negative control. Nonspecific binding and endogenous peroxidase activity were blocked using normal goat serum in 3% bovine serum albumin and 0.3% hydrogen peroxide in methanol, respectively. Sections were washed 3 times with phosphate buffered saline between steps and all steps were performed at room temperature. Labelling was enhanced with streptavidin-peroxidase (Vectastain Elite ABC kit, Vector Laboratories, Peterborough, UK) and visualized with diaminobenzadine.

## Results

### Ankle and Hip ACI patients

**Figure 1.**
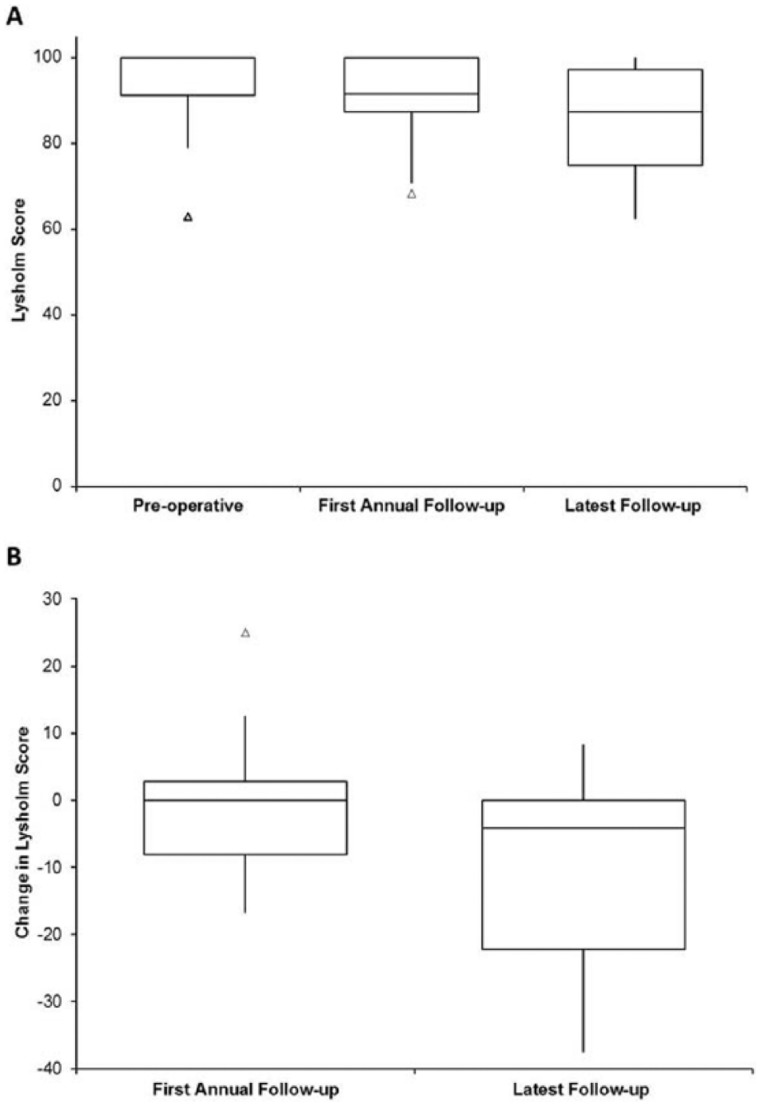
Box and whisker plots displaying (**A**) Lysholm scores for the following time points: preoperative, 13.7 ± 1.7 months postharvest (first annual review), and at 4.8 ± 1.8 years postharvest (latest follow-up) and (**B**) median difference in Lysholm scores at the first annual review and latest follow-up compared with preoperative scores. Lysholm scores at the first annual review (median 91.7) and at latest follow-up (median 87.5) were not significantly different to preoperative scores and neither was the difference in Lysholm score. The box and the horizontal line represent the interquartile range (IQR) and the median, respectively. Outliers are represented as a small triangle.

At a mean of 5.1 ± 8.5 days preoperatively (range 0-31 days), the median Lysholm score for all patients was 100 (interquartile range [IQR] 8.7). Median Lysholm scores at the first annual review (13.7 ± 1.7 months postharvest, range 12-18.3 months) and at 4.8 ± 1.8 years postharvest (range 1.2-7.8, termed “latest follow-up” from here on) were 91.7 (IQR 12.5) and 87.5 (IQR 22.2), respectively. Neither the first annual review nor the latest follow-up scores resulted in significantly different scores to the preoperative scores (**Fig. 1A**). The majority of patients were classified as having either an excellent or good Lysholm score at all 3 time points (**Table 1**). At latest follow-up, the median Lysholm score of the opposite knee was 100 (IQR 5.3) and significantly higher than the harvest knee (*P* = 0.0046). Women had a greater change in median score from the preoperative score compared with men at both the first annual follow-up (median change −4.2 and 0, respectively) and the latest follow-up (median change −16.7 and 0, respectively), but neither was significant, either between the time points or between the sexes.

**Table 1.** Classification of Lysholm Scores Preoperatively, at First Annual Review, and Latest Follow-up.

	Excellent (95-100), %	Good (84-94), %	Fair (65-83), %	Poor (≤64), %
Preoperatively	72	17	5.5	5.5
First annual review	42	37	21	—
Latest follow-up	33	22	39	5.5

**Figure 2.**
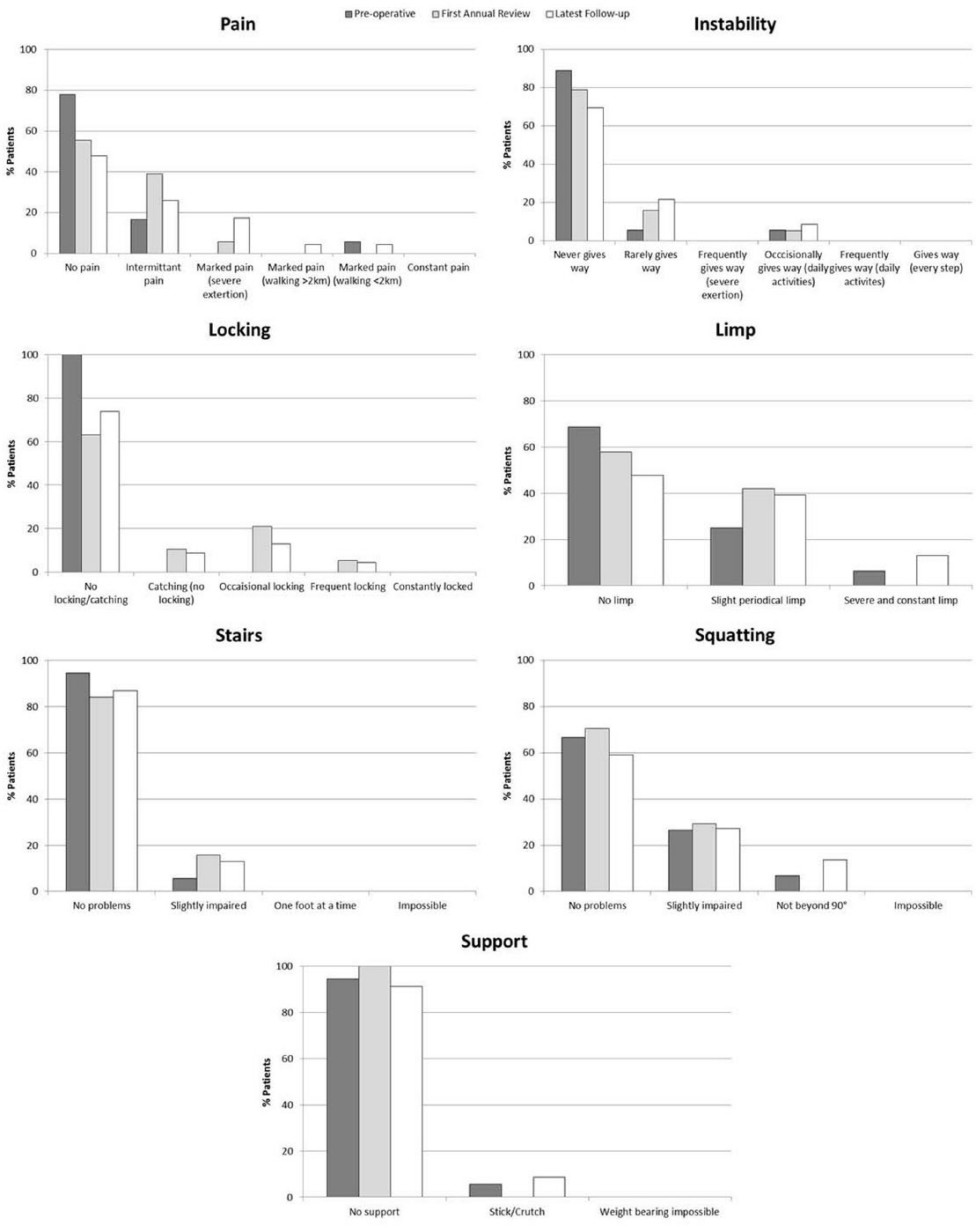
Histograms depicting the 7 different scoring parameters of the modified Lysholm score^13^ and the percentage of patients within each category preoperatively (dark gray bar), at the first annual review (light gray bar), and the latest follow-up (white bar). A significantly higher occurrence of pain was reported by patients at the latest follow-up compared with preoperatively (*P* = 0.05) and significantly more catching and locking sensations were reported at both the first annual review and latest follow-up (*P* = 0.004 and *P* = 0.019, respectively).

At the first annual review, Lysholm scores had decreased from preoperative scores in 44% of patients, increased in 25%, and remained the same in 31% of patients. The median change in Lysholm score from preoperative to first annual review was 0. By the latest follow-up, Lysholm scores had decreased from preoperative scores in 56% of patients, increased in 13%, and remained the same in 31% of patients. The median change in Lysholm score from preoperative to latest follow-up was −4.2 (**Fig. 1B**). From the Lysholm score, a significantly increased occurrence of pain was reported by patients at the latest follow-up compared with preoperatively (*P* = 0.05, **Fig. 2**). In addition, significantly more catching and locking sensations were reported by patients at both the first annual review and latest follow-up (*P* = 0.004 and *P* = 0.019, respectively, **Fig. 2**). Only 3 patients reported swelling of their knee. One patient reported a constantly swollen knee at the first annual review, but no swelling 6.3 years postharvest at latest follow-up. Two patients reported swelling only on severe exertion at 7.8 and 6.1 years follow-up.

**Figure 3.**
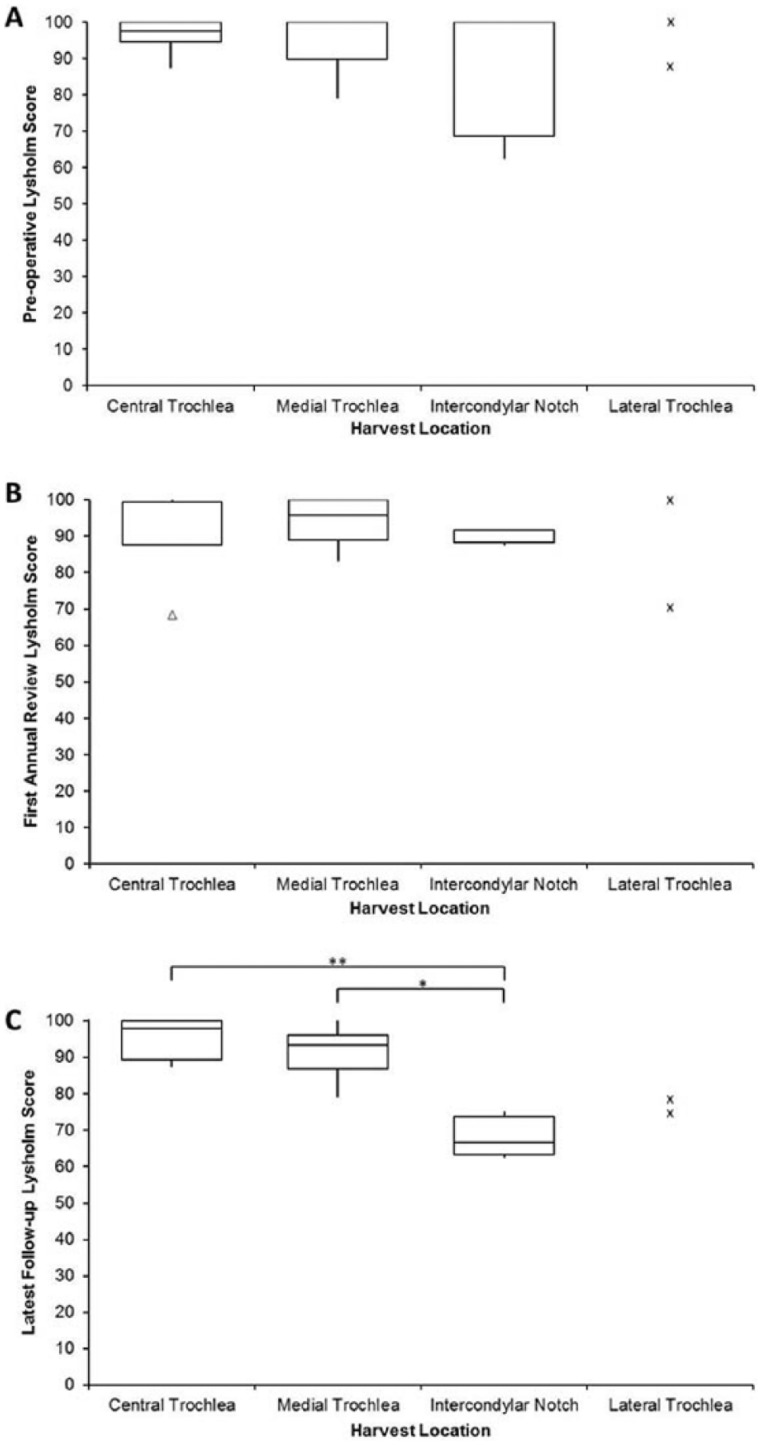
Box and whisker plots comparing Lysholm scores for different donor sites preoperatively (**A**), at first annual review (**B**), and at latest follow-up (**C**). Lysholm scores were not significantly different from each other preoperatively or at first annual review, but at the latest follow-up, the Lysholm score of patients who had chondral harvest from the intercondylar notch, had significantly lower scores than patients with harvests taken from the central trochlea (**P = 0.01) and the medial trochlea (*P = 0.02). Actual Lysholm scores for the lateral condyle at the 3 time points are represented as small crosses. The box and the horizontal line represent the interquartile range (IQR) and the median, respectively. Outliers are represented as a small triangle.

The influence of the location of the chondral harvest was considered; there was no significant difference between the mean ages of each harvest location group. The preoperative Lysholm score for harvests obtained from the central trochlea was 97.6 and 100 for both the medial trochlea and the intercondylar notch (**Fig. 3A**), with no significant differences in scores between the different harvest locations. At the first annual review, median Lysholm scores were lower, but not significantly, than preoperative scores for all regions; 87.5, 95.8, and 91.7 for the central trochlea, medial trochlea, and intercondylar notch, respectively (**Fig. 3B**). By the latest follow-up, Lysholm scores for the central trochlea had returned to preoperative levels (median 97.9, mean follow-up 5.5 ± 0.9 years, range 4-6.5 years). Lysholm scores for the medial trochlea group at the latest follow-up were maintained at 93.4 with a mean follow-up of 4.9 ± 1.6 years (range 3.4-7.8 years), while for the intercondylar notch group, the median Lysholm score was 66.7 with a mean follow up of 2.9 ± 2.1 years (range 1.2-6.1 years). These were significantly lower than for both the central and medial trochlea groups (*P* = 0.01 and *P* = 0.02, respectively, **Fig. 3C**). The 2 patients who had a chondral harvest from the lateral trochlea, had preoperative Lysholm scores of 87.5 and 100, first annual review scores of 70.8 and 100, and latest follow-up scores of 79.2 and 75.

There was no significant relationship between the site of chondral harvest and the weight of harvest taken (*P* = 0.33) or the Lysholm score at either first annual review or latest follow-up (*P* = 0.996 and *P* = 0.148, respectively). Patient’s age at ACI was also found not to correlate with either the preoperative or latest follow-up Lysholm scores (*P* = 0.59 and *P* = 0.12, respectively), nor were there any significant differences in either the first annual review or follow-up Lysholm scores between the sexes.

One patient in the series required a further knee arthroscopy 1.4 years postharvest after complaining of clicking and grinding behind the patella and reporting a Lysholm score of 68. At the stage I arthroscopy, this patient was noted by the surgeon to have a small grade III chondral defect on the trochlea; at this follow-up arthroscopy this defect was observed to be larger and was subsequently debrided. The chondral harvest site on the lateral trochlea was found to have healed with a good level of fill and with good integration with the surrounding native articular cartilage, but had a softer consistency. There were also some fronds present here, which were debrided. Five years postarthroscopy, this patient now reports a Lysholm score of 91.

### Additional Donor Site Study

**Figure 4.**
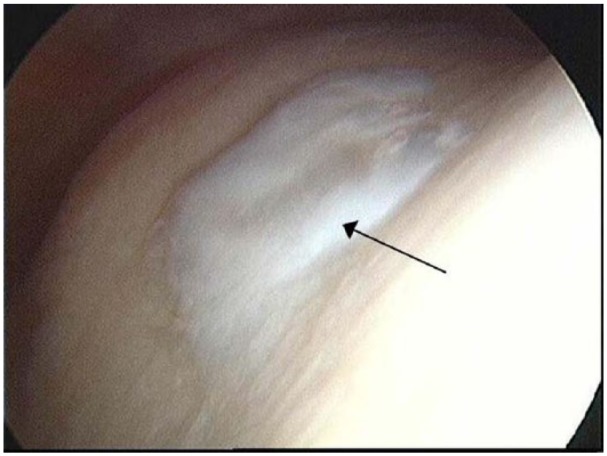
Representative arthroscopic image of the donor site in a patient 11 months post–cell implantation for autologous chondrocyte implantation **(**ACI) of the knee. The white portion in the center of the image (black arrow) is the repaired cartilage in the center of the trochlea, observed to be smooth and well integrated into the surrounding cartilage.

**Figure 5.**
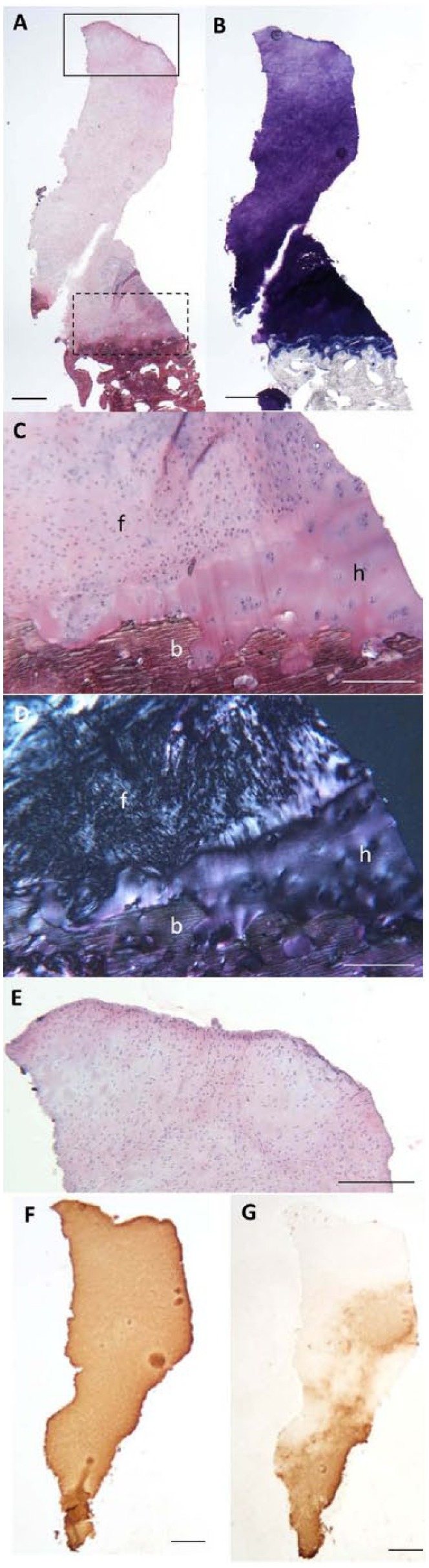
Representative histological images of a full depth repair tissue biopsy from the donor site in a patient 11 months post–cell implantation for autologous chondrocyte implantation (ACI) of the knee, stained with hematoxylin and eosin (H&E) (**A**) and toluidine blue (TB) (**B**). Histological analysis of the repair cartilage demonstrated good matrix metachromasia (B) and good integration into the subchondral bone (**C**, higher powered image of dashed-line box region in A). Polarized light revealed a mixture of hyaline cartilage and fibrocartilage (**D**, polarized image of C, f = fibrocartilage, h = hyaline cartilage, b = bone) also well integrated with each other. All biopsies demonstrated a good surface (**E**, higher power image of the solid line box region in A). Immunohistochemistry of sections demonstrate the widespread presence of type I collagen (**F**) but more restricted type II collagen in the lower region (G). Scale bars represent 500 µm (A, B, F, G) and 250 µm (C-E).

All 3 donor sites of the patients who underwent a follow-up arthroscopy after ACI of the knee, were seen by the surgeon to be well healed with smooth, white cartilage that appeared to be well integrated into the surrounding native hyaline cartilage (**Fig. 4**). The core biopsies obtained were full depth (**Fig. 5A**) with generally good matrix metachromasia (**Fig. 5B**). Under polarized light, 1 biopsy was observed to be hyaline cartilage and the other 2 biopsies were a mixture of both hyaline cartilage and fibrocartilage, the hyaline cartilage being at the base of the biopsy in both cases and well integrated into both the underlying subchondral bone and the upper fibrocartilaginous portion (**Fig. 5C** and **D**). All 3 biopsies had moderately good surface architecture (**Fig. 5E**) and generally good cell morphology with most cells being rounded and of a chondrocytic appearance. The mean ICRS II and OsScore histological scores for the biopsies taken from the donor site were 116.8 ± 10 and 8.7 ± 0.8, respectively. Immunohistochemical staining for collagen type I was detected throughout the full depth of the biopsies (**Fig. 5F**), whereas collagen type II was predominantly restricted to the hyaline portions of the biopsies, but could also be detected to a lesser amount in the fibrocartilaginous portions adjacent to and above this (**Fig. 5G**).

## Discussion

Autologous chondrocyte implantation has been used since 1994^5^ as treatment for cartilage defects with good long-term results.^20,21^ Little is known, however, of the effect of the chondral injury needed to source the chondrocytes for ACI on knee function. There are many reports on the use of osteochondral grafting for locations such as the ankle, where the donor site has been the knee; a few of these studies make reference to donor-site morbidity,^1-4,6,8,14,22-25^ but often is not a focused outcome of the study. Chondral harvest from the knee for the treatment of the ankle is rarely performed nowadays as most chondral harvests are taken from the joint to be treated^7,9,26^ for various reasons, including restricting surgical trauma to a second joint and both anatomical and biomechanical differences between knee and ankle cartilage.^27^ A recent study has also demonstrated that chondrocytes extracted from the margins of both grade III and IV osteochondral lesions in the knee are comparable to those from non-weightbearing areas of the joint currently used for chondral harvest.^28^ Adopting this type of approach would eliminate any potential for donor-site morbidity, but there are no long-term clinical data on the outcome of the use of debrided edge cartilage as a source of autologous chondrocytes.

To our knowledge, our study is the first to look at the effects of chondral harvest alone on knee function. We found that following a biopsy of approximately 270 mg of cartilage from the knee, the majority of patients maintained a good or excellent Lysholm score. Scores at 13.7 months postharvest were not significantly different to preoperative scores and were maintained at 4.8 years follow-up. Although our study did not assess the area of chondral harvest, we did record the mass of the tissue harvested and we did not find a significant correlation between it and change in knee function. In addition, we found no correlation between either the patient’s age or sex and knee function.

It is also worth noting that whilst none of the patients in our study had previously sought treatment for their knees prior to harvest, the preoperative Lysholm scores were not all 100, with some as low as 62.5 (where scores less than 83 are considered “fair” and scores less than 64 are considered “poor”).^16^ Two patients who received ACI for chondral defects in the hip, reported low preoperative Lysholm scores, which could be attributed to referred pain from the hip,^29^ particularly as the Lysholm score increased dramatically at the first annual review. This demonstrates how patient perception and clinical observation differ and do not always correlate; it also highlights the importance of comparing the difference in knee scores from pre- to postoperative rather than the postoperative score in isolation. Likewise, a reduced Lysholm score at the latest follow-up could possibly be due to either existing or newly developed degenerative changes within the joint and not actually related to the donor-site. However, our results demonstrate that the median Lysholm score at latest follow-up for the opposite knee to which the harvest was taken was significantly greater than the harvest knee, despite both knees having a median Lysholm score of 100 preoperatively. This, we believe, is a reflection on the spread of the data and the limited number of patients in this study. Indeed, some studies have refrained from using the Lysholm score as a measure of knee function when analyzing the effect of an osteochondral harvest for ankle mosaicplasty, as they believe that the score may be biased by the painful and functionally reduced ipsilateral ankle joint and hence mask the true knee score.^25^ We respect that the Lysholm score is not, in everyone’s opinion, the best score to use and there are several other scores that are also validated which are frequently used. However, the scores used in the present study are historical, some being collected up to 17 years ago when only Lysholm scores were collected. Despite this, the Lysholm score is recognised as a reliable measure for use in patients with chondral injuries.^30^

The first ACI procedures reported harvesting cartilage from “a minor load-bearing area of the upper medial femoral condyle.”^5^ One would assume this was to limit potential donor-site morbidity, although this particular rationale was not mentioned. A study using a biomechanical cadaveric model demonstrated the lowest contact pressures in the knee to be on the medial trochlea and thus recommended this location for future procedures requiring an osteochondral harvest to minimize donor site morbidity.^31^ Garretson *et al*.^31^ also suggest the “worse” place for an osteochondral harvest to be the central trochlea, due to having the highest contact pressures. Interestingly, in our study, we found patients who had a harvest obtained from either the central or the medial trochlea had significantly better Lysholm scores at latest follow-up than those where the harvest was taken from the intercondylar notch. Garretson *et al*.’s study did not include the intercondylar notch. Unfortunately, we were unable to statistically compare the effect of a chondral harvest from the lateral trochlea with the other locations included in this study due to lack of patients within this category, but, at the latest follow-up, both patients only had a “fair” Lysholm score indicating the lateral trochlea to be a less than optimal harvest location. However, the small number of patients involved in the study (*n* = 25) and even fewer numbers for individual locations (*n* = 2-10) is obviously a limiting factor in being able to make any statistically supported recommendations for an optimal location for chondral harvest.

It is difficult to make a direct comparison of donor site morbidity between osteochondral and chondral harvests due to the difference in the size of harvest, the types of tissue removed and the resulting defect created. One could speculate that perhaps an osteochondral harvest may in fact have an improved healing potential since they extend beyond the subchondral bone and thus encourage bleeding and entry of bone marrow stromal cells into the donor site, which may contribute to the repair process, much in the way that a standard microfracture procedure works. A chondral harvest, however, does not extend into the subchondral bone and so there is no such response stimulated. On the other hand, only 1 donor site is required for a chondral harvest for ACI, whereas procedures like mosaicplasty often result in multiple donor sites and have a greater overall donor area. Large areas of OATS harvests have previously been reported to correlate with a poorer Lysholm score at final follow-up.^4^

Macroscopically, all 4 donor sites (1 from knee harvest for ankle ACI and 3 from knee ACI patients) observed by arthroscopy in our study were found to have healed with good integration into the surrounding cartilage. One donor site in particular, which was noted to be of a softer consistency than the surrounding cartilage, could indicate the presence of a fibrocartilaginous-like tissue rather than a hyaline-like tissue. Microscopic examination of 3 repair tissue biopsies revealed 2 were a mixture of both hyaline cartilage and fibrocartilage while the other was solely hyaline cartilage. Previous studies have reported that following an osteochondral harvest, the donor site fills with a fibrocartilaginous tissue.^1^ It is possible that the hyaline cartilage observed microscopically, or at least some of it, could be residual native hyaline cartilage due to the nature of the gouge instrument used to harvest the initial cartilage for stage I and the “U-shape” of the defect left behind. However, there is evidence that the repair tissue formed at the treatment site following ACI matures with time^32,33^ and so maturation of the tissue formed in the donor site may also occur.

In conclusion, our study demonstrates that at a mean of 4.8 years after chondral harvest for ACI treatment, there was no significant change in median Lysholm knee scores compared to preoperative scores. These results suggest that there is no significant harvest site morbidity of the knee associated with harvesting cells for use in ACI, particularly when taken from the central or medial trochlea. Some patients, however, did report greater pain, catching and locking at their final follow-up, though whether this was due to donor site morbidity *per se* or injury or degeneration during the average 4.8-year postharvest follow-up time, is not possible to differentiate. However, one should carefully consider the location for chondral harvest as this has been shown to affect knee function long term. It is recommended that patients should be made aware that they may experience some functional deficit, which is likely to be transient. It is also vital that preoperative knee scores are measured, as patients who rate their knees as asymptomatic may have lower knee scores than the surgeon assumes. While this study demonstrates no significant donor-site morbidity and good healing of the chondral harvest site both macroscopically and microscopically, more in-depth studies with a larger cohort of patients are required to fully assess the effect of harvest location on knee function and both the *in vivo* appearance and the histological quality of the repair tissue formed within the donor site to fully assess donor site morbidity and understand the processes involved in what appears to be spontaneous cartilage regeneration.
